# CASK regulates CaMKII autophosphorylation in neuronal growth, calcium signaling, and learning

**DOI:** 10.3389/fnmol.2013.00027

**Published:** 2013-09-11

**Authors:** John M. Gillespie, James J. L. Hodge

**Affiliations:** School of Physiology and Pharmacology, University of BristolBristol, UK

**Keywords:** CASK, CaMKII, synaptic function, *Drosophila*, appetitive learning, calcium imaging, autophosphorylation

## Abstract

Calcium (Ca^2+^)/calmodulin (CaM)-dependent kinase II (CaMKII) activity plays a fundamental role in learning and memory. A key feature of CaMKII in memory formation is its ability to be regulated by autophosphorylation, which switches its activity on and off during synaptic plasticity. The synaptic scaffolding protein CASK (calcium (Ca^2+^)/calmodulin (CaM) associated serine kinase) is also important for learning and memory, as mutations in CASK result in intellectual disability and neurological defects in humans. We show that in *Drosophila* larvae, CASK interacts with CaMKII to control neuronal growth and calcium signaling. Furthermore, deletion of the CaMK-like and L27 domains of CASK (*CASK* β null) or expression of overactive CaMKII (T287D) produced similar effects on synaptic growth and Ca^2+^ signaling. CASK overexpression rescues the effects of CaMKII overactivity, consistent with the notion that CASK and CaMKII act in a common pathway that controls these neuronal processes. The reduction in Ca^2+^ signaling observed in the *CASK* β null mutant caused a decrease in vesicle trafficking at synapses. In addition, the decrease in Ca^2+^ signaling in CASK mutants was associated with an increase in Ether-à-go-go (EAG) potassium (K^+^) channel localization to synapses. Reducing EAG restored the decrease in Ca^2+^ signaling observed in CASK mutants to the level of wildtype, suggesting that CASK regulates Ca^2+^ signaling via EAG. CASK knockdown reduced both appetitive associative learning and odor evoked Ca^2+^ responses in *Drosophila* mushroom bodies, which are the learning centers of *Drosophila*. Expression of human CASK in *Drosophila* rescued the effect of CASK deletion on the activity state of CaMKII, suggesting that human CASK may also regulate CaMKII autophosphorylation.

## Introduction

CaMKII has been proposed to act as a molecular switch during increased neuronal activity, when increased Ca^2+^ levels stimulate CaMKII activity to induce the changes in synaptic strength that underlie learning. The ability of CaMKII to induce long lasting changes in synaptic strength has been shown to be dependent on CaMKII autophosphorylation at T286 (T287 in *Drosophila*). T287 autophosphorylation occurs in response to prolonged increases in Ca^2+^, which result in constitutively active CaMKII that is independent of Ca^2+^. This constitutive CaMKII activity has been suggested to be important for long-term potentiation (LTP) and memory in rodents (Giese et al., 1998; Hardingham et al., 2003; Sanhueza et al., 2011) and *Drosophila* (Park et al., 2002; Mehren and Griffith, 2004; Hodge et al., 2006; Malik et al., 2013). In *Drosophila*, CaMKII autophosphorylation is regulated at synapses by CASK, a membrane-associated guanylate kinase (MAGUK) that is kinase dead in *Drosophila* (Lu et al., 2003; Hodge et al., 2006).

Interactions of CASK with CaMKII can lead to inhibition of CaMKII activity through CaMKII autophosphorylation at a second pair of sites, T305/T306. This process results in reduced binding of CaMKII to CaM, which decreases kinase activation by Ca^2+^ and thereby prevents T287 autophosphorylation. CaMKII autophosphorylation at this site is important for long-term depression (LTD) and behavioral plasticity in mice (Elgersma et al., 2002; Pi et al., 2010) and *Drosophila* (Lu et al., 2003; Malik et al., 2013).

The function of CASK has also been studied in mice, and while CASK knock-outs are lethal due to a cleft palate phenotype, neurons cultured from these animals show abnormalities in glutamatergic synaptic release (Atasoy et al., 2007). However, the early lethality of these mice prevents the modeling of CASK function in behavior and disease.

*CASK* has two isoforms, a full-length *CASK* β isoform that contains the CaMK-like and L27 domains and PDZ, SH3, and guanylate kinase domains. The other isoform, *CASK* α, is short and contains only the common PDZ, SH3 and guanylate kinase domains and forms a molecule with structural homology to vertebrate MPP (Slawson et al., 2011). Previous characterization of CASK has focused on a large chromosomal deficiency that removes both forms of CASK and genes on either side of *CASK*. These deficiency flies exhibit reduced synaptic currents, defects in neuromuscular junction (NMJ) growth, decreased neurotransmitter release, decreased vesicle cycling and a loss of interaction with neurexin (Zordan et al., 2005; Sun et al., 2009; Chen and Featherstone, 2011).

To investigate the true role of CASK in *Drosophila*, a deletion of the full length *CASK* β isoform that resulted in viable adults was used. This *CASK* β null allele deletes only the CaMK-like and L27 domains that are unique to *CASK* β and leaves the short *CASK* α isoform and all flanking genes intact (Slawson et al., 2011). The *CASK* β null has specific deficits in larval locomotor behavior (Slawson et al., 2011) and adult sleep and place preferences (Donelson et al., 2012). Furthermore, *CASK* β is required for 3 h and long-term memory as measured in an adult aversive olfactory conditioning assay; this assay revealed that CASK is required in the α′/β′ subset of mushroom body neurons during memory formation (Malik et al., 2013).

In this study, we used *CASK* β null flies to more accurately analyze *CASK* synaptic function, as flanking loci are not deleted in this flies, which is the case for flies with the large chromosomal deficiency of the *CASK* region, which has been phenotypically characterized previously (Lu et al., 2003; Zordan et al., 2005; Hodge et al., 2006; Sun et al., 2009; Chen and Featherstone, 2011; Slawson et al., 2011). *CASK* β is shown to regulate CaMKII autophosphorylation during synaptic bouton growth and Ca^2+^ signaling. We also demonstrate that *CASK* β controls vesicle trafficking and the localization of EAG K^+^ channels to synapses. In *Drosophila* larvae, *CASK* β is required for appetitive associative learning and olfactory evoked Ca^2+^ responses in the mushroom body. Finally, we provide evidence that the human form of CASK seems capable of compensating for the loss of *Drosophila* CASK in regulating CaMKII autophosphorylation, suggesting a high level of conservation.

## Materials and methods

### Fly strains and genetics

All flies were grown at similar densities in bottles on standard medium at 22 ± 2°C. *CASK* β null, *uas-CASK (10,20 MI), uas-CaMKII, uas-CaMKII-T287D, uas-CaMKII-T287A* and *uas-TrpA1* (Lu et al., 2003; Pulver et al., 2009; Slawson et al., 2011) were kind gifts from Dr. Leslie Griffith (Brandeis University, US). *uas-EAG-RNAi* (stock #9126) and *uas-CASK-RNAi* flies (stock #104793, Malik et al., 2013) were obtained from the Vienna *Drosophila* Stock Center (VDRC). *MEF2-Gal4, 201Y-Gal4*, and *uas-cacophony-eGFP* (Ranganayakulu et al., 1996; Kawasaki et al., 2004; Thum et al., 2007) were from the Bloomington Stock Center. Wildtype flies [*Canton Sw*-, (*CSw*-)] were a kind gift from Dr. Scott Waddell (Oxford University, UK). *GCaMP3.1* flies (Tian et al., 2009) were a gift from Dr. Loren Looger (Janelia farm, VA, US). We are grateful to Dr. Hermann Aberle (Dusseldorf University, Germany) for *OK371-Gal4* flies (Mahr and Aberle, 2006). The *uas-human CASK* line has been described previously (Malik et al., 2013). *uas-GCaMP3* was meiotically recombined with *201Y-Gal4* flies on the 2nd chromosome and homozygosed. Similarly, *uas-GCaMP3, uas-TrpA1* and *OK371-Gal4, uas-GCaMP3* flies were generated by standard recombination crosses. All *CASK* mutants, *Gal4*, and *UAS* lines were outcrossed with the *CSw*- line for at least six generations prior to behavioral experiments.

### Immunohistochemistry

Third-instar larvae were dissected in HL3.1 [70 mM NaCl, 5 mM KCl, 10 mM NaHCO3, 115 mM sucrose, 4 mM MgCl, 5 mM trehalose, 1.5 mM CaCl_2_, and 5 mM HEPES (pH 7.3)] using standard techniques that have been used previously for these antibodies (Hodge et al., 2006; Cavaliere et al., 2012). The dissected larvae were fixed in 4% paraformaldehyde in HL3.1, permeabilized in HL3.1 with 0.1% triton X (HL3.1-tx), and then blocked in HL3.1-tx, 0.1% BSA, and 2% normal donkey serum (HL3.1-tx-BSA-NGS). Primary antibody incubations were performed as indicted with anti-pT287 CaMKII (Santa Cruz, rabbit, 1:150), anti-*Drosophila* CaMKII (Cosmo, mouse, 1:100), anti-HRP-FITC (Jackson ImmunoResearch Laboratories, 1:200), anti-*Drosophila* DLG-PDZ2 [rabbit, 1:1000 (Sherwood et al., 2004)] anti-*Drosophila* EAG [rabbit, 1:50 (Sun et al., 2004)], at 4°C in HL3.1-tx-BSA-NGS. After washing three times in HL3.1-tx, incubations were performed with anti-rabbit 405 nm, anti-rabbit 488 nm, or anti-mouse 648 nm fluorescently conjugated secondary antibodies (Alexa, Invitrogen, 1:400) in HL3.1-tx-BSA for 2 h at room temperature, after which the larvae were washed and mounted in glycerol and Vectorshield (Vector Laboratories).

### Imaging and quantification

All preparations within each immunohistochemistry experiment were processed in parallel, and images were acquired with identical settings using a Leica SP5 confocal microscope. Care was taken to maintain all intensity readings within the linear range below saturation. Measurements of mean intensity, bouton area, and quantification were performed manually with Volocity software (PerkinElmer) by drawing round type Ib and Is boutons on muscle 12. Because bouton number increases with muscle area (Schuster et al., 1996), bouton number was normalized to muscle area. The muscle area was determined by measuring the length and width of muscle 12.

For live *GCaMP3* experiments, larvae were dissected in HL3.1 and imaged essentially according to previously published protocols (Cavaliere et al., 2012). To prevent muscle contractions during NMJ imaging, HL3.1 was supplemented with 7 mM glutamate (HL3.1-glu). This concentration of glutamate desensitises the postsynaptic glutamate receptors without significantly affecting presynaptic Ca^2+^ signaling (Macleod et al., 2004). *uas-TrpA1, uas-GCaMP3/OK371-Gal4* larvae were stimulated by bath application HL3.1-glutamate at 35°C. Images were captured through a 63 × water immersion lens (*NA* = 0.9) on a Zeiss widefield Axio Examiner microscope, and images were taken at 4 frames per second. The average of the first 10 frames during periods of neuronal inactivity was calculated after subtraction of background fluorescence. *GCaMP3* intensity values were normalized to this baseline intensity. Ventral ganglion imaging was performed in similar manner using HL3.1 without glutamate on a Zeiss widefield Axio Examiner microscope (10 × water immersion lens *NA* = 0.3), and images were taken at 10 frames per second.

Similar to previous studies (Asahina et al., 2009), larval mushroom body imaging was performed by first cutting off the heads of the larvae in HL3.1. The salivary glands and digestive tract were removed to allow visualization of the brain. A small hole was made in a strip of PVDF membrane, which was then placed across a custom-made airflow chamber that allowed odor to be puffed onto the larval head. The dissected head of the larvae was placed into this hole so that the tip of the head was on one side of the PVDF membrane and the brain was on the other side. Low melt agarose (1%) in HL3.1 was applied over the top of the brain to prevent the media from covering the tip of the larvae's head and was allowed to set at 4°C for 1 min. HL3.1 was then added to the top of this agarose to allow for imaging of GCaMP3 in the mushroom body calyx through a 63× water immersion lens (*NA* = 0.9) on a Zeiss widefield Axio Examiner microscope. Images were acquired using identical exposure times at 10 frames per second. After recording for approximately 1 min, during which time a constant air flow was delivered via an aquarium pump, the air was the passed through a chamber containing a piece of tissue soaked in hexyl acetate (Sigma, 1:10 in mineral oil) for approximately 10 s before the odor-free air was applied. The part of the calyx specifically activated by the application of odor was selected as the region of interest, and the mean background-subtracted intensities for each time point were calculated for this region. The ratios of the mean intensity at each time point to the intensity at baseline were calculated.

### FM1-43 imaging

FM1-43 loading of motor neuron terminals at larval NMJs was performed as described previously (Sun et al., 2009). Wandering third-instar larvae were dissected in Ca^2+^-free HL3 as above. The larvae were then incubated with 1 μ M FM1-43 in 90 mM KCl and HL3 with 1.5 mM Ca^2+^ for 10 min. FM1-43 loading was terminated by washing the larvae five times for 10 min with Ca^2+^-free HL3. A stack of images of the labeled vesicles was acquired for quantification using the 63× objective (*NA* = 0.9) of a Zeiss widefield Axio Examiner microscope, and all settings remained constant between preparations. The mean labeling intensities of approximately 10 boutons per NMJ on muscle 12 were determined using Volocity.

### Larval appetitive learning

Larval learning experiments were performed as previously described (Chen et al., 2011). Approximately 100 flies were allowed to lay eggs for 12 h in bottles containing standard media and maintained at 25°C. These eggs were then allowed to develop for 5 days, and the wandering third-instar larvae were then separated from the food by floating in a 15% sucrose solution prior to being washed in running tap water. These larvae were then used immediately in the larval learning experiments. Larval training was performed using 9 cm petri dishes containing approximately 10 ml 1% agarose dissolved in ddH_2_O with or without 2 M fructose. Odors were presented in an odor cup made from the top of a 500 μl thin-walled micro-centrifuge tube. The bottom of the tube (1 cm from the top) was cut away and replaced with the lid of another micro-centrifuge tube. Holes in the lid were then made using a pair of sharp forceps heated with a Bunsen burner. A small piece of blotting paper containing 20 μl of either hexyl acetate or benzaldehyde (Sigma) diluted 1:10 with mineral oil was placed in the odor cup. Odor cups containing the stimulus that had been conditioned with reinforcement (CS+) were placed 1 cm from the edge on either side of the petri dish containing fructose (unconditioned stimulus, US). The larvae were allowed to crawl in this dish for 5 min before being transferred to the petri dish that contained pure agarose and the stimulus that had been conditioned without reinforcement (CS−) for a further 5 min. This process was repeated three times. For testing, the larvae were then placed in a 7 mm wide stripe in the middle of a pure agarose plate with the CS+ and CS− odors on opposite sides of the dish. After 3 min the larvae on either side of dish and in the 7 mm wide middle strip were counted. A performance index (PI) was then calculated according to the following equation:

$$\text{Performance index}=\left({\#}_{CS+}-{\#}_{CS-}\right)\text{​}/{\#}_{\text{Total}}$$

The odors representing the *CS*+ and *CS*− were then swapped, and the test was repeated to produce a second PI that was averaged with the first PI to yield an n of 1.

To measure fructose acuity, larvae were placed in the middle of a petri dish that contained pure agarose gel in one half and agarose gel containing 2 M fructose in the other half. Larvae were placed in the middle of the fructose acuity test plate and allowed to crawl for 5 min after which a PI was calculated according to the following equation:

$$\text{Performance index}=\left({\#}_{\text{Fructose}}-{\#}_{\text{Pure}}\right)\text{​}/{\#}_{\text{Total}}$$

Odor acuity control experiments were performed by placing odors on one side of a pure agarose petri dish and allowing larvae to crawl for 3 min after which the larvae on either side of dish and in the 7 mm wide middle strip were counted to calculate a preference index using the following equation:

$$\text{Performance index}=\left({\#}_{\text{Odour}}-{\#}_{\text{Pure}}\right)\text{​}/{\#}_{\text{Total}}$$

### Statistical analysis

All statistical analyzes was performed in Prism (GraphPad). All data were analyzed with unpaired two-tailed Student's *t*-tests or One-Way ANOVAs with Bonferroni *post-hoc* testing where appropriate. We ensured that the variances did not differ significantly between groups. For each experiment, the details of the statistical analyzes and N's can be found in the figure legends. In all figures, error bars represent the SEM. No asterisk indicates *p* > 0.05, ^*^ indicates *p* < 0.05, ^**^ indicates *p* < 0.01 and ^***^ indicates *p* < 0.001.

## Results

### The CaMK-like and L27 domain-containing isoform of cask regulates synaptic terminal growth

**Figure 1:**
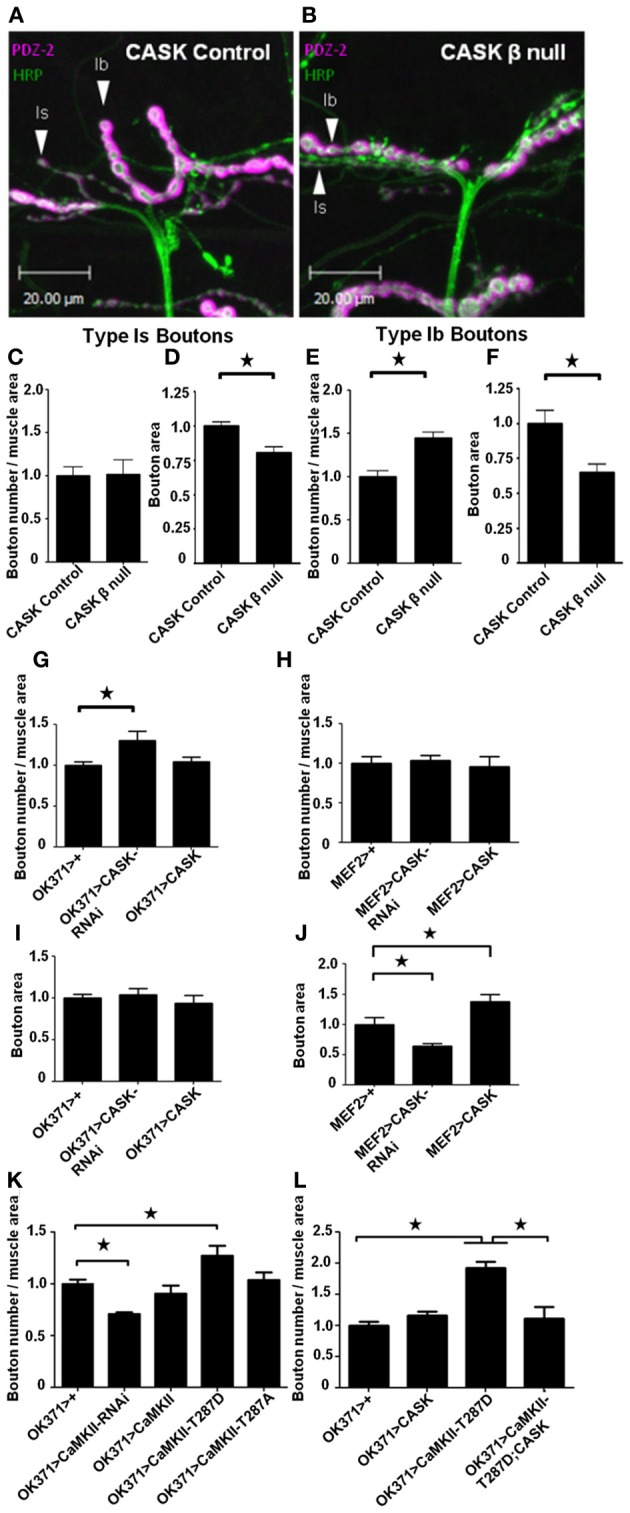
**CASK and CaMKII regulate synaptic morphology**. Representative images of muscle 12 of *Drosophila* third-instar larval neuromuscular junctions (NMJ) visualized with the presynaptic marker anti-HRP (green) and the postsynaptic marker anti-DLG (PDZ1-2, magenta). NMJs of larvae with precise **(A)** and imprecise **(B)** excisions of *CASK* (*CASK* β null) that remove the CaMK-like and L27 domain-containing version of *CASK* (Slawson et al., 2011). **(C)** Compared to controls, the total number of small (type 1s) boutons normalized to muscle area did not change (*p* > 0.05, *n* = 6). **(D)** However, the area of the 1s boutons was significantly (*n* = 6, *p* < 0.05) decreased in *CASK* β null larvae. Regarding, the larger (Type 1b) boutons, *CASK* β deletion caused an increase (*p* < 0.05, *n* = 6) in synapse number **(E)** and a decrease in size **(F)** (*p* < 0.05, *n* = 6). **(G)** Pre-synaptic (*OK371-Gal4*) reduction in *CASK* was sufficient to cause an increase (*p* < 0.05, *n* = 6) in 1b bouton number, but had no effect on bouton area **(I)**. Postsynaptic (*MEF2-Gal4*) overexpression of *CASK* **(J)** caused an increase (*p* < 0.05, *n* = 6) in 1b bouton area **(H)** without affecting bouton number (*p* > 0.05, *n* = 6). Presynaptic overexpression of the constitutively active CaMKII phosphomimic (T287D) transgene (Park et al., 2002) caused an increase (*p* < 0.05, *n* = 6) in synapse number **(K)**. Conversely, reducing presynaptic CaMKII activity (*CaMKII-RNAi*) reduced (*p* < 0.05, *n* = 4) 1b bouton number. **(L)** Presynaptic (*OK371-Gal4*) overexpression of T287D increased (*p* < 0.05, *n* = 6) type 1b bouton number, while CASK overexpression alone had little effect compared to controls. Co-expression of CaMKII-T287D with CASK (*OK371-Gal4>uas-CASK/uas-CaMKII-T287D*) returned type 1b bouton number to control levels (*p* < 0.05, *n* = 5). All data in **(C–F)** were analyzed with unpaired *t*-tests. All data in **(G–L)** were analyzed with One-Way ANOVA and Bonferroni *post-hoc* testing. In this and all subsequent figures, error bars represent the SEM. No asterisk indicates *p* > 0.05, ^*^ indicates *p* < 0.05, ^**^ indicates *p* < 0.01 and ^***^ indicates *p* < 0.001.

To determine the role of the long CASK isoform in control of synaptic morphology and the regulation of CaMKII autophosphorylation, we characterized the NMJs of homozygous *CASK* β wandering third-instar mutant larvae. *Drosophila* larval NMJs have a stereotyped pattern in which identified motor neurons form specific types of boutons (type Ib, Is, II and III boutons) on particular muscles (Hoang and Chiba, 2001). On muscle 12, motor neuron MN12-Ib forms type Ib boutons that are large (3–10 μm) and contain glutamatergic synapses. Motor neuron MNISNb/d-Is (RP5) terminates on muscle 12 and forms type Is boutons that are also glutamatergic, but these boutons are smaller in size (2–4 μm) (Hoang and Chiba, 2001). Larvae were dissected and co-stained with anti-HRP (green), which labels the presynaptic neuronal membrane (Jan and Jan, 1982), and anti-DLG-PDZ2 (magenta), a marker for the postsynaptic density (PSD) and sub-synaptic reticulum (Sherwood et al., 2004). Using pre- and post-synaptic markers, we quantified the effect of CASK and CaMKII on synaptic bouton morphology. Compared to controls (Figure 1A), *CASK* β null NMJs appeared to contain more boutons that were smaller in size (Figure 1B). Quantification of the number of type 1s boutons revealed similar numbers between control and *CASK* β null larvae (Figure 1C). However, the sizes of the *CASK* β null boutons were smaller than those of controls (Figure 1D). *CASK* β null NMJs contained more type 1b boutons (Figure 1E) that were also smaller in size than those of the controls (Figure 1F). To explore whether CASK was functioning pre- or post-synaptically to bring about these changes in synaptic terminal morphology, we used a transgene to overexpress *CASK* (Hodge et al., 2006) and an *RNAi* transgene specific for *CASK* (Malik et al., 2013) that were either expressed in motor neurons [*OK371-Gal4*, (Mahr and Aberle, 2006)] or muscle [*MEF2-Gal4*, (Ranganayakulu et al., 1996)]. The reduction in presynaptic *CASK* by *RNAi* knockdown was sufficient to cause the increase in type 1b boutons (Figure 1G) that was also observed in *CASK* β null larvae but had no effect of bouton area (Figure 1I). The change in bouton area observed in *CASK* β null larvae seemed to be mediated by a postsynaptic function of CASK, as muscle expression of *CASK-RNAi* reduced type Ib bouton area, and CASK overexpression increased type 1b bouton area (Figure 1J), but had no effect on bouton number (Figure 1H).

To address whether these CASK-dependent synaptic changes were brought about via CASK's regulation of CaMKII (Lu et al., 2003; Hodge et al., 2006), we tested whether transgenic manipulation of CaMKII would recapitulate these synaptic defects. Reducing *CASK* is known to result in increased CaMKII-T287 autophosphorylation (Hodge et al., 2006). Consistent with this, we found that expressing CaMKII-T287D presynaptically phenocopied the increase in type 1b boutons observed after deletion of CaMK-like and L27 domain-containing CASK or the presynaptic reduction of CASK. Conversely, *RNAi* mediated reduction in presynaptic CaMKII activity (Ashraf et al., 2006) caused a reduction in bouton number (Figure 1K).

Phosphorylation at TT306/7 is known to occur after T287 autophosphorylation and reduce the kinase activity of CaMKII by between 20 and 80% (Hanson and Schulman, 1992; Jama et al., 2009; Coultrap et al., 2010). Overexpression of CASK causes an increase in CaMKII-T306 T307 autophosphorylation (Hodge et al., 2006) that reduces kinase activity. Therefore, we co-expressed CASK concurrently with CaMKII-T287D presynaptically and found this rescued the increase in bouton number observed after presynaptic T287D expression alone, confirming that CASK and CaMKII act together in a common presynaptic pathway that controls synaptic growth (Figure 1L).

### The CaMK-like and L27 domain-containing isoform of cask regulates the switch of synaptic CaMKII to calcium independence

**Figure 2:**
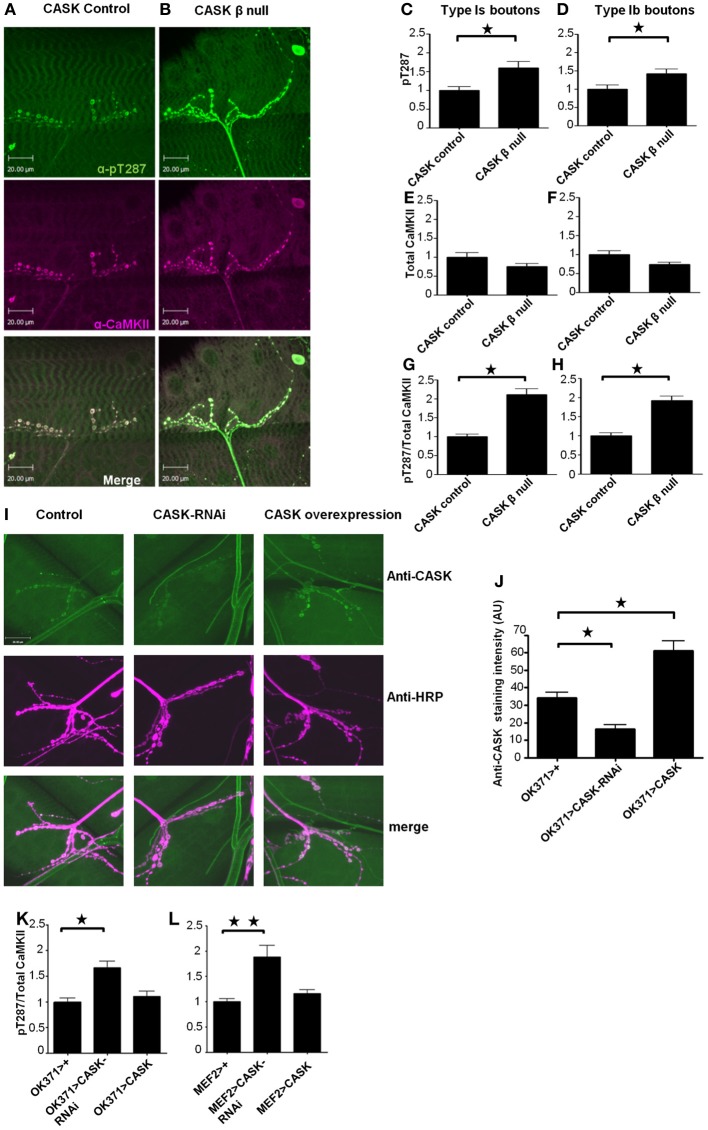
**The CaMK-like and L27 domain-containing version of CASK regulates CaMKII autophosphorylation at synapses**. Compared to control **(A)** *CASK* β null larvae **(B)** exhibited increased levels of synaptic CaMKII autophosphorylation at T287 [as visualized in green using a phospho-specific antibody to CaMKII-T287 (Hodge et al., 2006)] relative to total synaptic CaMKII [visualized in magenta using an antibody raised against CaMKII (Hodge et al., 2006)]. Overlapping expression is shown in white. Removal of the CaM-Kinase and L27 domains of CASK caused an increase (*p* < 0.05) in pT287 at 1s **(C)** and 1b **(D)** boutons. The total amount of CaMKII localized at synapses was not affected (*p* > 0.05) by the removal of CASK **(E,F)**. The ratio of pT287 to total CaMKII **(G,H)** also showed a significant (*p* < 0.05) increase in the *CASK* β null larvae. **(I)** Representative images of CASK (green) and HRP (magenta) stained NMJs from control larvae, larvae overexpressing *CASK-RNAi* in their motor neurons and larvae overexpressing *CASK* in their motor neurons. **(J)** Quantification of the effect of *CASK* knockdown or overexpression on anti-CASK staining showed that genetic manipulation of CASK significantly changed the amount of CASK present at the NMJ (*p* < 0.05). Reductions in CASK either pre- **(K)** or post-synaptically **(L)** caused increases (*p* < 0.05) in CaMKII (T287) autophosphorylation. All data in **(C–H)** were analyzed by unpaired *t*-tests (*n* = 6). All data in **(J–L)** were analyzed by One-Way ANOVA with Bonferroni *post-hoc* tests (*n* = 6).

While it has previously been shown that deletion of the *CASK* gene results in dysregulation of CaMKII autophosphorylation (Hodge et al., 2006), these mutant flies also have heterozygous deletions of genes on either side of the *CASK* locus. Furthermore, the shorter isoform of CASK (*CASK* α) was also deleted in these studies (Slawson et al., 2011). Therefore, to determine the role of the CaMK-like and L27 domain-containing isoform of CASK in the regulation of synaptic CaMKII autophosphorylation, we used a CaMKII-T287 phospho-specific antibody (Figures 2A,B, green). We found that the mean bouton pT287 CaMKII staining intensity was greater in *CASK* β null larvae for the type 1s (Figure 2C) and 1b boutons (Figure 2D) than in controls. While this result may be due to *CASK* β regulating CaMKII autophosphorylation, CASK could also conceivably have a scaffolding role that localizes CaMKII to synapses, or alternatively, CASK could increase the density of CaMKII present in the boutons by reducing bouton size. To address these questions, we co-stained NMJs with an antibody to total CaMKII (Figures 2A,B, magenta). The total level and spatial distribution of CaMKII was unchanged in *CASK* β null synapses compared to controls (Figures 2E,F), confirming that the amount of T287 autophosphorylation per synaptically localized CaMKII molecule had indeed increased (Figures 2G,H) and that CASK was not required for localizing CaMKII to synaptic bouton sites. To determine whether CASK was regulating CaMKII autophosphorylation by a pre- or post-synaptic mechanism, we knocked down *CASK* on either side of the synapse. The effect of these genetic manipulations on CASK levels were verified by immunohistochemistry, which showed that CASK knockdown in motor neurons reduced CASK expression at the NMJ by 52% compared to controls (Figures 2I,J). We found that either motor neuron (Figure 2K) or muscle (Figure 2L) expression of *CASK-RNAi* was sufficient to cause a switch to the Ca^2+^-independent constitutively active (pT287) state of CaMKII.

### Cask and CaMKII act in a common pathway regulating activity-dependent calcium signaling

**Figure 3:**
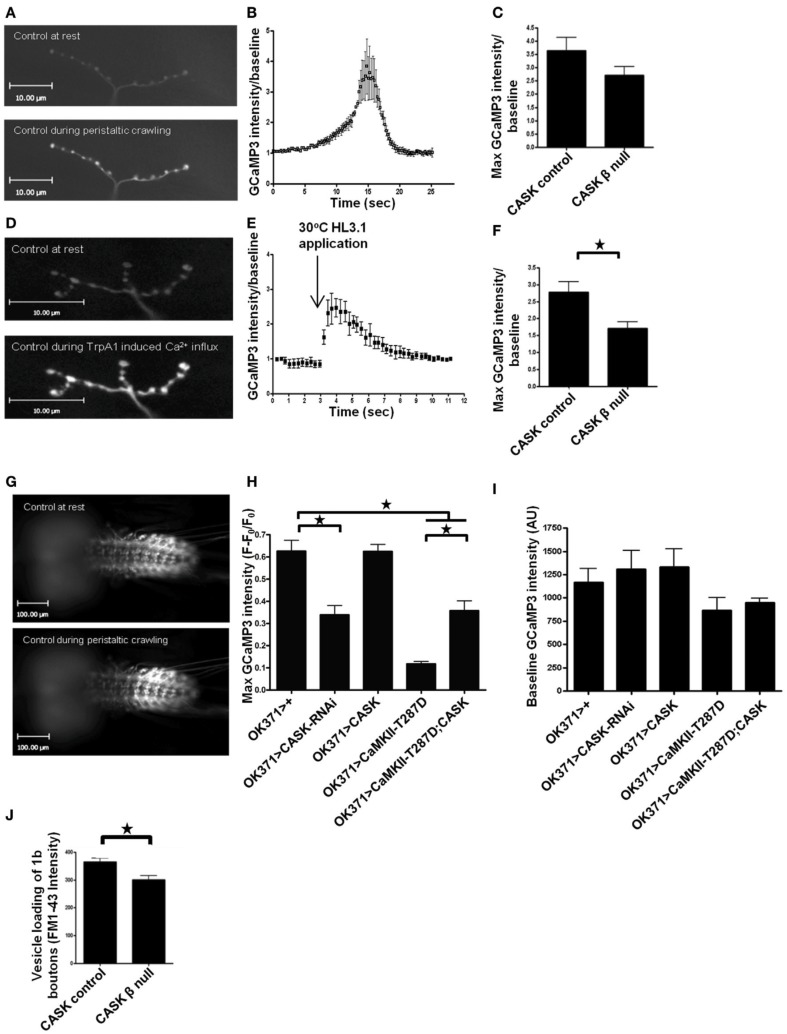
**The CaMK-like and L27 domain-containing version of CASK regulates calcium signaling and vesicle cycling**. Compared to resting synapses, there is an increase in Ca^2+^ signaling during peristaltic crawling, and this increase is reported by the genetically encoded Ca^2+^ indicator GCaMP3, which was expressed in motor neurons, as shown in representative images of larval muscle 12 NMJs **(A)** This increase can be observed as a Ca^2+^-dependent increase in GCaMP3 fluorescence intensity compared to baseline **(B)** *CASK* β deletion non-significantly reduced this signal **(C)** (*p* > 0.05, *n* = 3, unpaired *t*-test). Application of heated (30°C) HL3.1 saline to acutely heat-activate the synaptic terminals of motor neurons expressing TrpA1 (*OK371-Gal4>uas-TrpA1/uas-GCaMP3*) caused a robust Ca^2+^ influx at muscle 12 NMJs **(D)** that could also be observed on a time trace showing maximum GCaMP3 intensity/baseline **(E)**. This increase in Ca^2+^ signaling was reduced (*p* < 0.05, *n* = 3, unpaired *t*-test) in the *CASK* β null background **(F)**. The increase in Ca^2+^ signaling during peristaltic crawling also occurred in the segmentally arranged motor neuron cell bodies in the ventral ganglion **(G,H)**. **(H)** Reduction in CASK (*OK371-Gal4>uas-CASK-RNAi/uas-GCaMP3*) caused a reduction (*p* < 0.05, *n* = 6, One-Way ANOVA with a Bonferroni *post-hoc* test) in peak GCaMP3 fluorescence relative to baseline compared to control. Similarly, increased expression of T287D reduced peak Ca^2+^ levels (*p* < 0.05, *n* = 4). CASK co-overexpression (*OK371-Gal4>uas-GCaMP3/uas-CASK;uas-CaMKII-T287D*) partially rescued the decrease in peak Ca^2+^ level compared to larvae with increased expression of T287D alone (*p* < 0.05, *n* = 4), but this peak level was still lower than observed in controls (*p* < 0.05, *n* = 4). **(I)** No significant differences were observed in baseline GCaMP3 levels between genotypes (*p* > 0.05, *n* = 4–6, One-Way ANOVA with a Bonferroni *post-hoc* test). **(J)** To investigate the synaptic consequences of the reduction in Ca^2+^ in presynaptic terminals, we measured vesicle recycling and found that there was a significant reduction (*p* < 0.05, *n* = 3, unpaired *t*-test) in loading of FM1-43 in *CASK* β null larvae compared to controls.

To further understand how CASK and CaMKII may be altering neuronal function *in vivo*, we expressed the genetically encoded calcium reporter GCaMP3 (Tian et al., 2009; Cavaliere et al., 2012) in these motor neurons to study activity-dependent changes in Ca^2+^ signaling (Figure 3). Transient increases in motor neuron Ca^2+^ are likely to control the movement of the larvae. Larvae crawl by peristaltic contractions of muscles in the tail segment that are followed by sequential posterior to anterior contraction of each segment (Cattaert and Birman, 2001). We reasoned that if there were endogenous peristaltic contractions of the larval preparation, then these contractions should be accompanied by increases in Ca^2+^ signaling at the NMJs of the contracting body wall segment (Figures 3A–C). As *CASK* β null larvae have a number of deficits in locomotion (Slawson et al., 2011), we sought to determine whether there were any changes in NMJ Ca^2+^ signaling that occurred during these peristaltic contractions. Although there was a reduction in this Ca^2+^ signaling, it was not significant (Figures 3A–C); this finding is likely due to the substantial movement artifacts, which increased the standard deviation of the recorded responses in this preparation. Therefore, we co-expressed the heat-activated ion channel TrpA1, which, when exposed to 30°C, causes a large depolarization and activation of these neurons (Pulver et al., 2009). Heat activation of TrpA1 robustly stimulated these motor neurons resulting in a large increase in synaptic Ca^2+^ signaling (Figures 3D–F), and while process induced some movement, the movement could be more easily controlled for than that during peristaltic contraction. The increase in peak Ca^2+^ induced by heat activation of TrpA1 was greatly reduced when the CaMK-like and L27 domain-containing version of CASK was absent in the *CASK* β null larvae (Figure 3F).

The cell bodies of the motor neuron NMJs that were imaged in the previous set of experiments are located in the ventral ganglion, where they are arranged in a segmentally repeated pattern such that motor neurons present posteriorly project to muscles located in the most posterior segments. Imaging of the ventral ganglion showed sequential activation of motor neurons characterized by waves of activation that moved from posterior to anterior segments and represented fictive crawling (Figure 3G). This increase in motor neuron cell body Ca^2+^ was quantified (Figure 3H). Reduction of CASK in these motor neurons reduced peak Ca^2+^ signaling but resulted in no significant change at baseline (Figure 3I). Similarly, increased CaMKII-T287D in these neurons also produced a similar reduction in peak Ca^2+^ level, and this deficit was partially reversed by co-expression of CASK (Figure 3H). This finding is again consistent with CASK and CaMKII autophosphorylation acting in the same pathway that regulates both activity-dependent Ca^2+^ signaling and synaptic bouton morphology.

We were interested in the functional consequences of the reduction in synaptic Ca^2+^ signaling observed in the *CASK* β null background. Previous work has shown that CASK is involved in neurotransmitter release and vesicle cycling in *Drosophila* synapses (Sun et al., 2009) because a chromosomal deficiency of CASK reduces vesicle trafficking. To determine whether this reduction was due to the CaMK-like and L27 domain-containing version of CASK, we measured activity-dependent vesicle recycling by loading FM1-43 dye into vesicles at the NMJ of control and *CASK* β null larvae and stimulating the synapses with 90 mM KCl for 10 min. We found that removal of *CASK* β was sufficient to reduce vesicle trafficking (Figure 3J).

### Downstream mechanisms of cask that regulate calcium signaling

**Figure 4:**
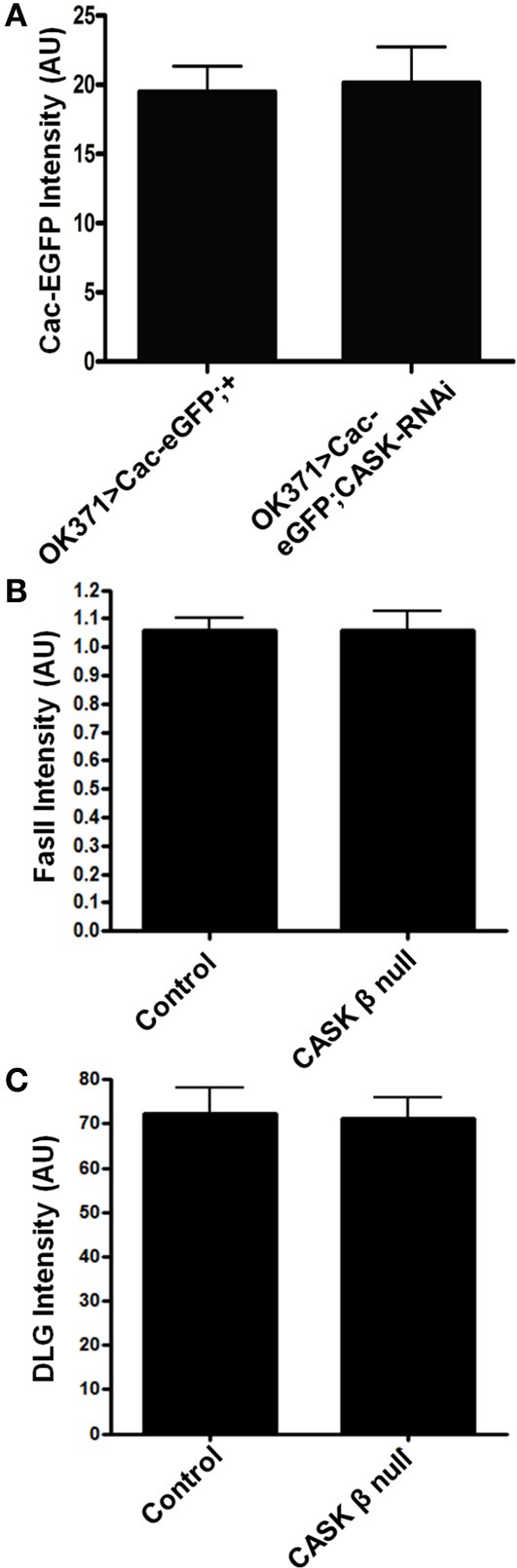
**CASK does not regulate the localization of Cav2, FasII, or DLG to synapses. (A)** *CASK* knockdown had no effect on the intensity of Cac-EGFP at the NMJ compared to control (*p* > 0.05, *n* = 6, unpaired *t*-test). **(B)** *CASK* β deletion did not significantly affect the intensity of FasII staining at the NMJ (*p* < 0.05, *n* = 6, unpaired *t*-test). **(C)** *CASK* β deletion did not significantly affect the intensity of DLG staining at the NMJ (*p* < 0.05, *n* = 6, unpaired *t*-test).

*CASK* knockdown has previously been shown to reduce the localization of CaV2.2 Ca^2+^ channels to the plasma membrane in mouse hippocampal primary cell culture (Samuels et al., 2007). We investigated whether *CASK* knockdown in *Drosophila* larvae reduced the localization of a GFP-tagged CaV2 Ca^2+^ channel [*uas-Cacophony-eGFP*, (Kawasaki et al., 2004)] to the NMJ and found no significant effect (Figure 4A). CaMKII-T287D expression has previously been shown to phosphorylate the scaffolding protein DLG, which reduces the localization of this protein to synapses (Koh et al., 1999). This loss of synaptic DLG reduces FasII localization and results in an increase in bouton number. However, quantification of mean bouton DLG (Figure 4B) or FasII (Figure 4C) antibody-specific staining intensity at the NMJ showed no effect of CASK β deletion.

**Figure 5:**
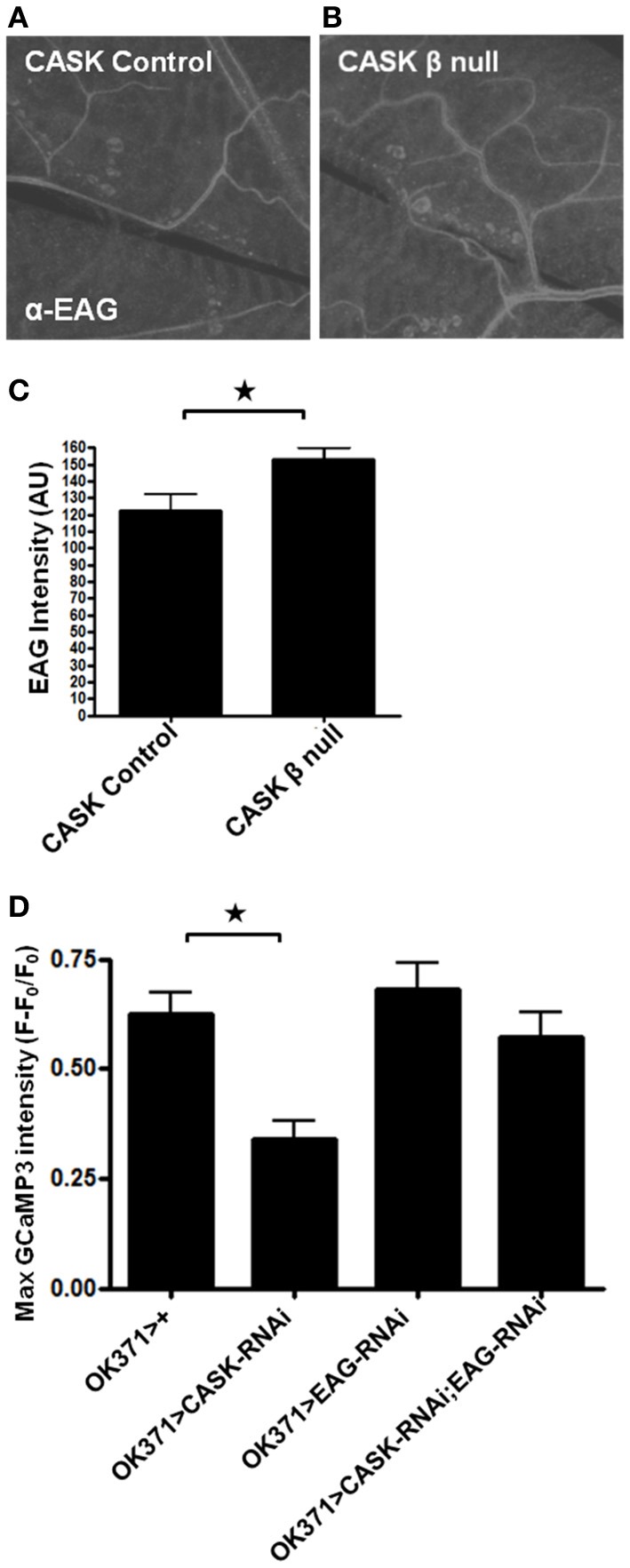
**Signaling downstream of CASK in calcium signaling and synaptic growth. (A,B)** Representative images of EAG staining at the NMJ in control and *CASK* β null larvae. **(C)** *CASK* β deletion significantly increased the intensity of EAG staining at the NMJ (*p* < 0.05, *n* = 6, unpaired *t*-test). **(D)** GCaMP3 imaging of the ventral ganglion during fictive crawling shows that the decrease in peak Ca^2+^ level induced by *CASK* knockdown was rescued by *EAG* knockdown. *EAG* knockdown alone did not significantly increase Ca^2+^ signaling (*p* > 0.05, *n* = 5–6, One-Way ANOVA with a Bonferroni *post-hoc* test).

CaMKII-T287D is known to increase K^+^ conductance in larval *Drosophila* muscles and to cause an increase in action potential failures in motor neurons (Park et al., 2002). These effects may be mediated through CaMKII phosphorylation of the K^+^ channel EAG at T787, which increases EAG K^+^ current in oocytes (Wang et al., 2002). Mutation of this amino acid to a phospho-blocking residue also reduces localization of the channel to the plasma membrane (Marble et al., 2005). However, as CASK coexpression with EAG in oocytes increases EAG localization to the plasma membrane, this finding presents a divergence in the potential effects of CASK on EAG function; CASK may reduce EAG function via CaMKII inhibition or increasing EAG function via a direct interaction. To investigate whether CASK's regulation of EAG in *Drosophila* is involved with the decrease in Ca^2+^ signaling in motor neurons, NMJs were stained (Figure 5A) with an EAG-specific antibody (Sun et al., 2004). Compared to controls, there was an increase in EAG localization to the NMJ in *CASK* β null larvae (Figures 5B,C). This finding is consistent with the notion that CASK regulates Ca^2+^ signaling via an interaction with CaMKII to alter neuronal excitability via EAG. To explore whether this interpretation is correct, *EAG-RNAi* was co-expressed with *CASK-RNAi* to investigate whether EAG reduction would prevent the CASK knockdown-induced impairment of Ca^2+^ signaling. Compared to control, knock down of *EAG* was sufficient to rescue the effects of *CASK* knockdown (Figure 5D). This finding is consistent with the notion that CASK acts with EAG in a common pathway to regulate Ca^2+^ signaling.

### Cask in the mushroom body is required for associative memory formation and odor-evoked calcium responses

**Figure 6:**
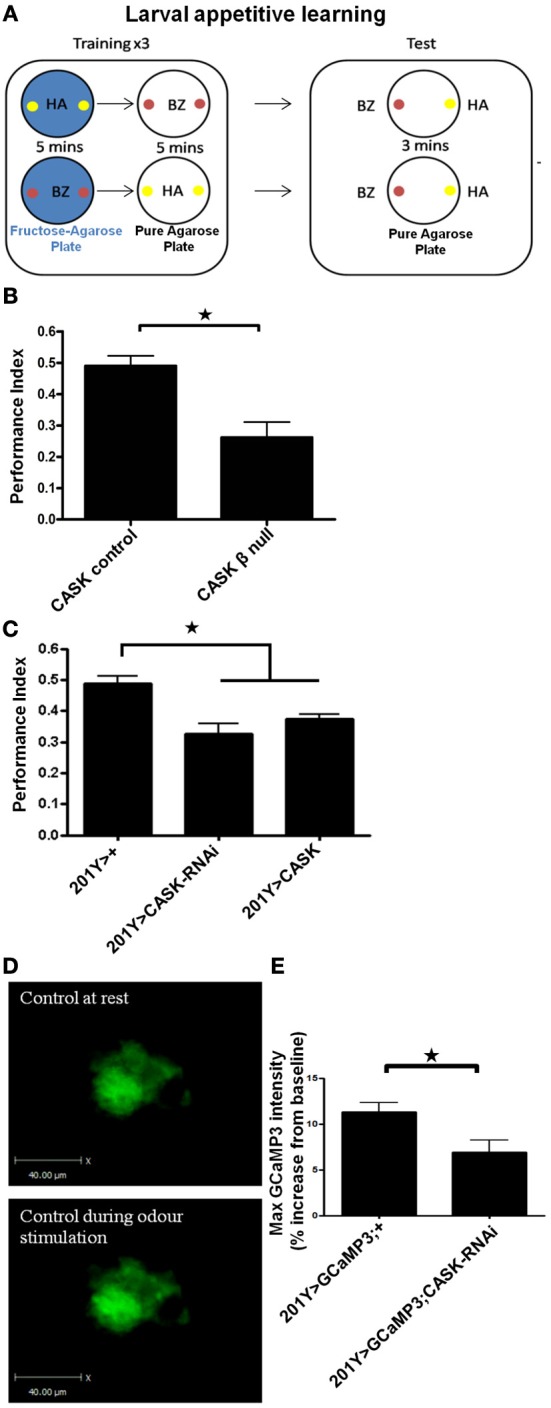
**The CaMK-like and L27 domain-containing version of CASK is required for appetitive learning. (A)** The role of CASK in associative memory was assessed by a larval olfactory-sugar reward conditioning protocol (Chen et al., 2011) described by the cartoon. The large rounded square on the left depicts the training phase of the protocol. Larvae were sequentially placed on petri dishes, represented by the large black circles, that contained either pure agarose (white filled circles) or 2 M fructose agarose (blue filled circles, the unconditioned stimulus, US). At the same time, the larvae were exposed to one of two neutral odors (the conditioned stimulus, CS). In the example given (top row) the larvae received 5 min of hexyl acetate on fructose-agarose (HA, small yellow filled circles, CS+) and then 5 min of benzaldehyde on agarose alone (BZ, small red filled circles, CS−). This training cycle was repeated 3 times before the test phase (depicted by the large rounded circle on the right) in which the larvae were allowed to move toward one of the two odors on pure agarose. The larvae showed learning by going toward HA (CS+). A performance index was calculated as the number of larvae that went to the CS+ minus the number of larvae that went to CS− divided by the total number of larvae. Bottom row, the odors representing the CS+ and CS− were swapped to produce a second performance index that was averaged with the reciprocal of the performance index to give an n of 1. **(B)** *CASK* β deletion caused a reduction (*p* < 0.05, *n* = 4, unpaired *t*-test) in learning compared to controls. **(C)** Compared to controls, learning was decreased (*p* < 0.05, *n* = 6, One-Way ANOVA with a Bonferroni *post-hoc* test) by both *CASK* knockdown and overexpression in the γ –lobe neurons of the mushroom body. **(D)** Representative images of GCaMP3 expressed in the γ –lobe neurons of the mushroom bodies at rest and during odor application. **(E)** Odor-evoked Ca^2+^ responses were reduced (*p* < 0.05, *n* = 6, unpaired *t*-test) by *CASK* knockdown in the mushroom body.

**Figure 7:**
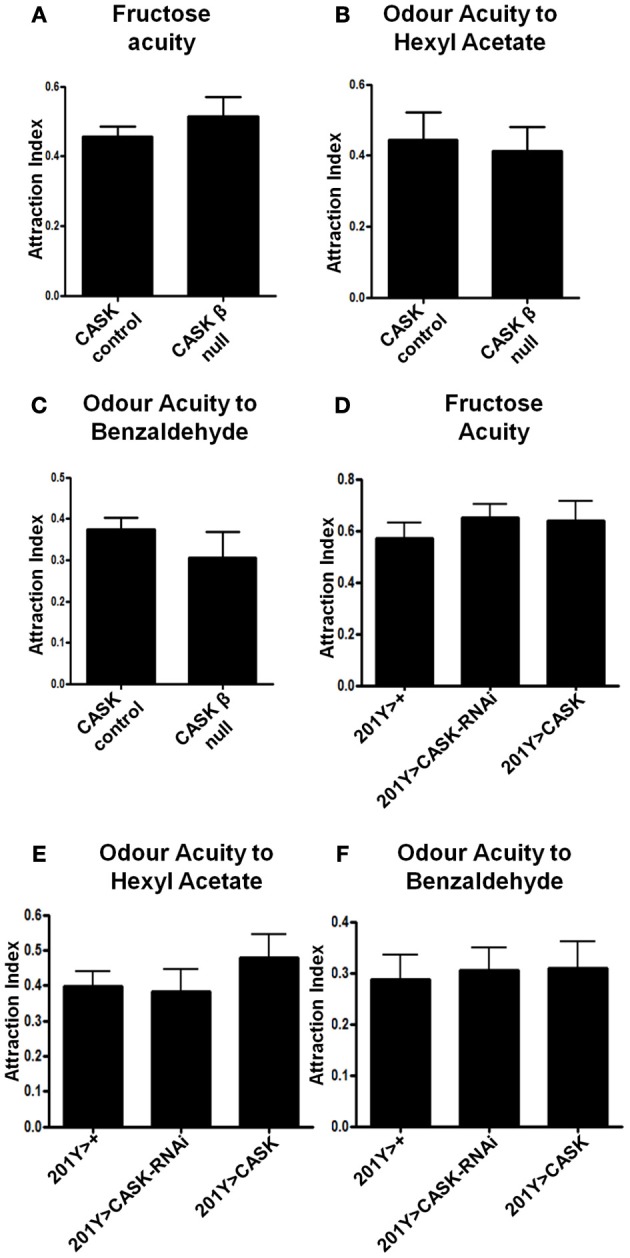
**CASK does not regulate odor or fructose acuity. (A–C)** *CASK* β deletion did not affect odor or fructose acuity compared to controls (*p* > 0.05, *n* = 3, unpaired *t*-test). **(D–F)** *CASK* knockdown or overexpression in the γ –lobe neurons of the mushroom body had no significant effects on odor or fructose acuity compared to controls (*p* > 0.05, *n* = 3, One Way ANOVA with a Bonferroni *post-hoc* test).

These changes in activity-dependent neuronal Ca^2+^ signaling and synaptic morphology are consistent with a role of the CaMK-like and L27 domain-containing isoform of CASK in synaptic plasticity. To explore the functional consequence of *CASK* β-mediated changes in neural function, we tested *CASK* β null larvae for appetitive associative learning (Figure 6A) using adapted previously published protocols (Tully and Quinn, 1985; Chen et al., 2011). *CASK* β null larvae were deficient in the ability to associate an odor with a positive reward (fructose, Figure 6B). To determine which neurons mediated this deficit in memory caused by the loss of CASK function, we specifically knocked down *CASK* in the mushroom bodies using *201Y-Gal4* (Pauls et al., 2010). This *Gal4* line allows for the expression of transgenes under *uas* control in the γ neurons of the larval mushroom body, the function of which is essential for larval appetitive learning (Pauls et al., 2010). Bidirectional changes in CASK expression in the γ neurons led to memory impairments (Figure 6C), indicating that the correct level of CASK in the mushroom body is required for memory formation. We expressed GCaMP3 under *201Y-Gal4* to investigate the responses of these olfactory memory neurons to odor application. *CASK* knockdown reduced odor-induced Ca^2+^ signaling (Figures 6D,E). The *CASK* mutant genotypes did not exhibit changes in odor acuity or the ability to sense fructose reward (Figure 7). This assay requires larvae to move toward these stimuli; thus, locomotor impairment does not prevent *CASK* mutant larvae from participating in this task.

### Human cask expression rescues the effect of cask β deletion on CaMKII autophosphorylation

**Figure 8:**
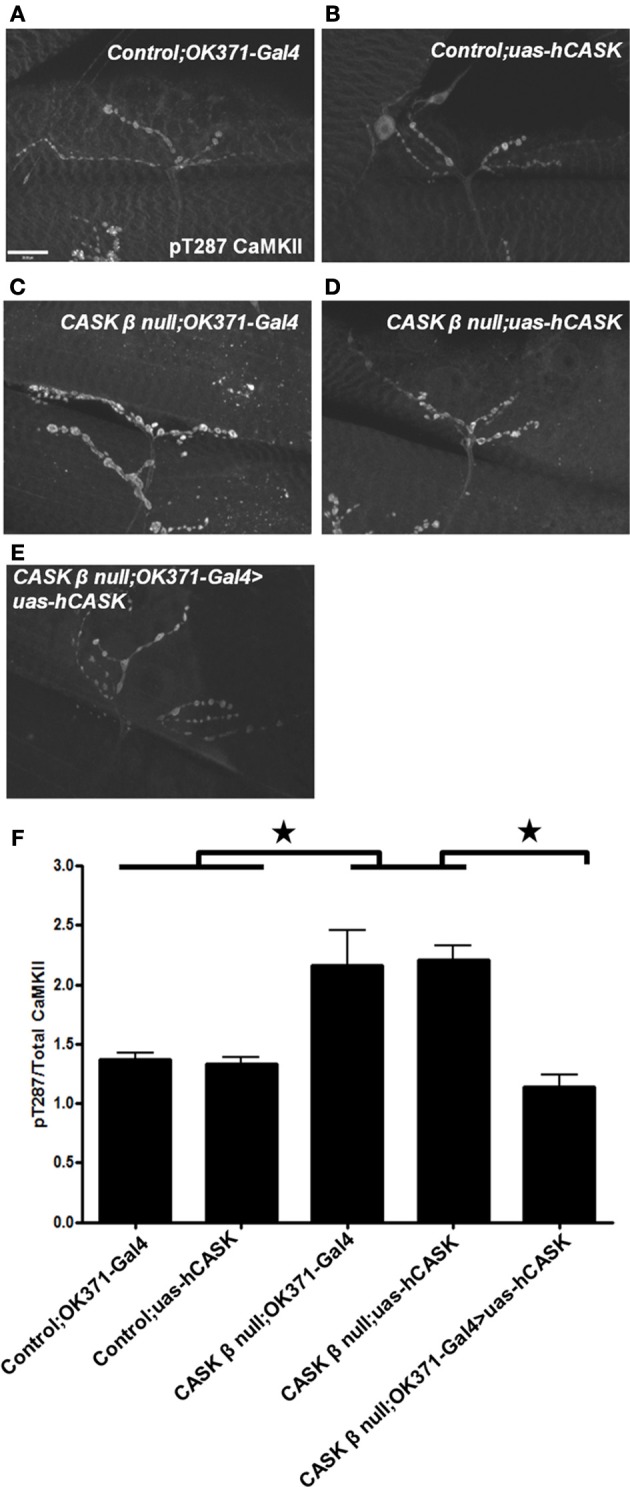
**Human *CASK* expression rescues the effect of *CASK* β deletion on CaMKII autophosphorylation**. Representation images of pT287 CaMKII-stained NMJs from **(A)** *OK371-Gal4/+* control larvae **(B)** *uas-human CASK/+* control larvae **(C)** *CASK* β null; *OK371-Gal4* mutant larvae **(D)** *CASK* β null; *uas-human CASK* mutant larvae **(E)** *CASK* β null; *OK371-Gal4>uas-human CASK* rescue larvae. **(F)** *CASK* β null; *uas-human CASK* and *CASK* β null; *OK371-Gal4* mutant larvae had significantly elevated pT287 CaMKII levels compared to *uas-human CASK* or *OK371-Gal4* larvae (*p* < 0.05). Motor neuron expression of human *CASK* in a *CASK* β null background significantly reduced pT287 CaMKII levels to levels that were indistinguishable from wildtype (*p* > 0.05, *n* = 6, One Way ANOVA with a Bonferroni *post-hoc* test).

As the amino acids of *Drosophila* CASK and CaMKII are highly homologous with their human homologs [74 and 79%, respectively, (Cho et al., 1991; Hsueh, 2006)], it is possible that human CASK would also interact with CaMKII. To investigate if human CASK can regulate *Drosophila* CaMKII autophosphorylation, human CASK was expressed in *Drosophila* motor neurons in a *CASK* β null background. Immunohistochemistry was performed to examine changes in pT287 CaMKII at the NMJ and showed that the expression of human CASK rescued the dysregulation of CaMKII autophosphorylation induced by deletion of CASK β (Figures 8A–F).

## Discussion

CaMKII autophosphorylation is a central mechanism in synaptic plasticity and associative memory formation in mammals and *Drosophila* (Giese et al., 1998; Lisman et al., 2002; Park et al., 2002; Hardingham et al., 2003; Mehren and Griffith, 2004; Hodge et al., 2006; Sanhueza et al., 2011; Malik et al., 2013). Therefore, the finding that CASK can regulate CaMKII autophosphorylation suggests that this mechanism may have a role in the cognitive deficits induced by CASK mutation in humans (Froyen et al., 2007; Najm et al., 2008; Piluso et al., 2009; Tarpey et al., 2009). To explore this hypothesis, we investigated the physiological significance of the regulation of CaMKII autophosphorylation by CASK. We show for the first time that this interaction regulates synaptic growth, behaviorally induced Ca^2+^ signaling and appetitive learning. Furthermore, although it has previously been shown in *Drosophila* that CASK increases CaMKII TT306/7 phosphorylation to reduce T287 autophosphorylation (Lu et al., 2003; Hodge et al., 2006), we demonstrated that human CASK expression in *Drosophila* also regulates CaMKII autophosphorylation. These findings suggest that dysregulation of CaMKII autophosphorylation is potentially a mechanism that underlies neurological disorders resulting from CASK mutations.

### Cask regulates synaptic bouton morphology and rescues the effects of CaMKII overactivity

The role of CASK in regulating synaptic bouton growth at the NMJ has been studied, but the results are inconsistent. One study showed an increase in the total number of type 1 boutons at the NMJ of *Drosophila* larvae containing a large chromosomal deficiency that removed both *CASK* β and *CASK* α and produced heterozygous deletions of genes on either side of *CASK* (Sun et al., 2009). However, a second study that also used these *CASK* deficiency larvae showed that the total number of type 1 boutons did not change, although this study reported a reduction in the number of active zones (Chen and Featherstone, 2011). We have attempted to resolve these inconsistencies by studying larvae with a deletion that specifically removed the CaMK-like and L27 domain-containing isoform of CASK (Slawson et al., 2011). We show that deletion of the CASK β isoform alone caused an increase in type I synaptic bouton growth. Remodeling of presynaptic boutons is known to occur in the rat mossy fiber system after spatial learning, which suggests that control of presynaptic growth is likely to be important for the formation of long-lasting memories (Holahan et al., 2006). As we showed that CASK influences the control of synaptic growth by regulating CaMKII activity, this role of CASK may be a mechanism by which CASK dysfunction impairs learning and memory.

### Cask rescues the effects of CaMKII overactivity on calcium signaling

As Ca^2+^ signaling induced by neuronal activity is important in the control of synaptic strength (Unni et al., 2004), the reduction of Ca^2+^ signaling and synaptic growth resulting from CASK knockdown is consistent with the notion that CASK knock down causes deficits in synaptic plasticity and, hence, learning. These results suggest that CASK regulates Ca^2+^ signaling through CaMKII. At the *Drosophila* NMJ, CaMKII primarily decreases synaptic activity, as neuronal expression of CaMKII-T287D is known increase the probability of the failure of evoked responses (Park et al., 2002). Consistently, we found that CaMKII-T287D expression also reduced Ca^2+^ signaling. When both *CASK* and *CaMKII-T287D* were co-expressed, Ca^2+^ signaling was rescued compared to *T287D* expression alone, again suggesting that these molecules are interacting in a pathway that controls Ca^2+^ signaling. A similar reduction in GCaMP3 response has been observed with *CASK* knockdown or *CaMKII-T287D* expression in the adult mushroom body (Malik et al., 2013). As both reductions in CASK and *CaMKII-T287D* expression in motor neurons induce locomotor defects in larvae, the reduction of Ca^2+^ signaling induced by *CASK* knockdown or *CaMKII-T287D* expression may therefore, also be involved in this locomotor phenotype. We also showed that the reduction in presynaptic Ca^2+^ observed in the *CASK* β null terminals results in a reduction in activity-dependent vesicle trafficking. This finding is consistent with a role of CASK in control of synaptic vesicle release as suggested by studies in a range of other systems (Butz et al., 1998; Zordan et al., 2005; Hsueh, 2006; Spangler et al., 2013).

### Cask is required for larval appetitive learning and odor-induced calcium signaling in the mushroom body

Using a larval appetitive olfactory learning assay, we demonstrated that CASK is required for associative learning and this function was localized to γ neurons in the mushroom body. This neuronal type is essential for larval appetitive learning (Pauls et al., 2010). We also showed that the Ca^2+^ signals induced by odor application are reduced by CASK knockdown in these neurons. As odor-induced Ca^2+^ signaling is likely to be critical for the induction of appetitive learning (Thum et al., 2007), this may be a mechanism by which CASK mutation impairs learning and memory.

One of the mechanisms through which CASK mutation may affect Ca^2+^ signaling is the regulation of EAG K^+^ channels. CaMKII and EAG act in a common pathway that is required for *Drosophila* synaptic and behavioral plasticity (Griffith et al., 1993, 1994). Overexpression of the EAG K^+^ channel BEC1 in the mouse forebrain impairs spatial working memory and tetanus-induced LTP, and the BEC1 heterozygous knockout exhibited significantly enhanced spatial working memory (Miyake et al., 2009). We provide evidence that CASK's regulation of EAG K^+^ channels, via CaMKII, regulates Ca^2+^ signaling, suggesting that this pathway may be important in learning and memory.

Expression of human CASK in the mushroom body has recently been shown to be able to completely restore the 3 h aversive memory deficit of *CASK* β null adults to wildtype levels (Malik et al., 2013). As human CASK was shown in the present study to regulate CaMKII autophosphorylation in *Drosophila*, this study, along with previous work, validates the use of *Drosophila* to study CASK and CaMKII in the healthy brain and in disease.

Mutations in human CASK can lead to a number of neurological diseases including FG syndrome 4, X-linked mental retardation with or without nystagmus and intellectual disability and microcephaly with pontine and cerebella hypoplasia (Froyen et al., 2007; Najm et al., 2008; Piluso et al., 2009; Tarpey et al., 2009). Therefore, investigating the mechanisms by which CaMKII autophosphorylation is regulated by CASK may be important for understanding the aetiologies of diseases involving these two proteins.

### Conflict of interest statement

The authors declare that the research was conducted in the absence of any commercial or financial relationships that could be construed as a potential conflict of interest.
